# Supplementary material for: Short-term exposure to air pollution on peripheral white blood cells and inflammation biomarkers: a cross-sectional study on rural residents
Source: BMC Public Health. 2024 Jun 26;24:1702. doi: 10.1186/s12889-024-19116-2 (PMC11201365; doi:10.1186/s12889-024-19116-2)
Supplement: Supplementary file 1 — Supplementary Material 1 [file 12889_2024_19116_MOESM1_ESM.docx]

**Short-term exposure to air pollution on peripheral white blood cells and inflammation biomarkers: a cross-sectional study on rural residents**

Yishu Yang, Hui Wu, Yuling Zeng, Fei Xu, Shuaiqi Zhao, Ling Zhang, Zhen An, Huijun Li, Juan Li, Jie Song, Weidong Wu*

Henan International Collaborative Laboratory for Health Effects and Intervention of Air Pollution, School of Public Health, Xinxiang Medical University, Xinxiang, Henan Province 453003, China

* Corresponding author:

Weidong Wu, PhD

School of Public Health,

Xinxiang Medical University,

Xinxiang, Henan Province 453003, China

Email: Wdwu2013@126.com

**Abstract**

Effects of short-term exposure to ambient air pollution on systemic immunological and inflammatory biomarkers in rural population have not been adequately characterized. From May to July 2021, 5816 participants in rural villages of northern Henan Province, China, participated in this cross-sectional study. Blood biomarkers of systemic inflammation were determined including peripheral white blood cells (WBC), eosinophils (EOS), basophils (BAS), monocytes (MON), lymphocytes (LYM), neutrophils (NEU), neutrophil-lymphocyte ratio (NLR), and serum high-sensitivity C-reactive protein (hs-CRP). The concentrations of ambient fine particulate matter (PM_2.5_), PM_10_, nitrogen dioxide (NO_2_), carbon monoxide (CO), and ozone (O_3_) were assessed up to 7 days prior to the blood draw. A generalized linear model was used to analyze the associations between air pollution exposure and the above-mentioned blood biomarkers. Significantly positive associations were revealed between PM_2.5_, CO and WBC; CO, O_3_ and LYM; PM_2.5_, PM_10_, SO_2_, CO and NEU; PM_2.5_, PM_10,_ SO_2_, CO and NLR; PM_2.5_, PM_10_, SO_2_, NO_2_, CO, O_3_ and hs-CRP. Meanwhile, negative associations were found between SO_2_ and WBC; PM_2.5_, PM_10_, NO_2_, CO, or O_3_ and EOS; PM_2.5_, SO_2_, or CO and BAS; SO_2_, NO_2_ or O_3_ and MON; PM_2.5_, PM_10_, SO_2_, or NO_2_ and LYM. Moreover, men, individuals with normal body mass index (BMI), current smokers, and those older than 60 years were found vulnerable to air pollution effects. Taken together, short-term exposure to air pollution was associated with systemic inflammatory responses, providing insight into the potential mechanisms for air pollution-induced detrimental systemic effects in rural residents.

**Keywords**: Air pollution; Inflammation; Cross-sectional study; Neutrophil-lymphocyte ratio

**1. Introduction**

Air pollution is a major environmental threat to human health worldwide and has been shown to be associated with a wide range of adverse health outcomes. Previous epidemiological studies have shown that exposure to air pollution leads to a variety of adverse health outcomes, including cardiorespiratory and autoimmune diseases, and can even increase mortality in the population [1–4]. Many pathophysiological alterations, such as inflammation, oxidative stress, and immunological disorders, have been attributed to air pollution exposure [5, 6]. Documented studies have indicated that air pollutants are capable of stimulating airway epithelial cells and immune cells, resulting in airway epithelial damage and detrimental effects beyond the lung, including systemic immunological and inflammatory responses [7–10].

One of the primary systemic inflammatory responses is the stimulation of the haematopoietic system to release leukocytes and platelets into the circulation. Previous studies have shown that leukocytes are a reliable predictor for air pollution-induced short- and long-term health effects [11-13]. Epidemiological investigations have discovered the relationships between short-term exposure to ambient air pollutants and the alterations of peripheral white blood cells in urban populations, such as peripheral white blood cells (WBC), eosinophils (EOS), basophils (BAS), monocytes (MON), lymphocytes (LYM), and neutrophils (NEU), neutrophil/lymphocyte ratio (NLR) [13–15]. In particular, NLR responds to the dynamic changes in neutrophils and lymphocytes and can be applied to quantify the dynamic action of cellular innate and adaptive immune responses, being considered as a new perspective marker of and a valid indicator of systemic inflammation [17, 18]. Thus far, the association of short-term air pollution exposure with NLR has been less characterized, with no evidence available for rural populations. In addition, high-sensitivity C-reactive protein (hs-CRP), a simple, rapid and cost-effective biomarker of systemic inflammation [18], has been widely utilized to assess health effects of short-term air pollution exposure due to its high sensitivity, but the existing evidence appears inconsistent [19–21].

Xinxiang is located north of Henan province, one of the most populous provinces and the top region by rural population in China, and is one of the regions in the country with severe air pollution [22]. Noteworthy, the population in this present study was living in a typical and modern rural area with traditional agriculture and community-managed industries. This represents the new trend of village development (rural revitalization) in modern China. Thus, the findings from this study could apply to other rural areas in Henan province or part of other provinces. Therefore, it is of practical significance to evaluate health consequences of air pollution on rural dwellers in this developing regions. Given that systemic inflammation is a common and essential event in the pathogenesis of many diseases, examination of blood biomarkers for systemic inflammation is crucial to identify early alterations of adverse health effects and take measures to prevent progression of diverse noncommunicable chronic diseases. It should also be noted that examination of white blood cells as well as soluble biomarkers of systemic inflammation was an indispensable part of the funded national project which supported this present study. In this context, the aim of this present study was to evaluate the effects of short-term exposure to ambient air pollution on peripheral white blood cells and serum biomarker for systemic inflammation in the rural population of Xinxiang, and to provide clues for design of preventive and therapeutic strategies.

**2. Methods**

**2.1 Study participants**

5816 participants were finally recruited in rural villages of Xinxiang from May to July 2021. Trained investigators employed standardized questionnaires to gather individual data, such as demographic characteristics, lifestyle habits, and disease history. Smoking, drinking, and physical activities were specified as previously reported [23]. Herein, smoking was defined as a minimum of one cigarette a day for more than 6 months; drinking as consuming alcohol 12 or more times annually, and physical activity levels as high, moderate, and low based on the physical activity-metabolic equivalent. The disease status of the participants was designated by the Charlson Comorbidity Index (CCI) [24]. Body mass index (BMI) was calculated as the ratio of body weight in kilograms(kg) divided by the square of height in meters (m^2^). The Ethics Committee of Xinxiang Medical University for Human Studies authorized this investigation (XYLL-2016242). Moreover, the informed consent was given willingly and in writing by each participant.

**2.2. Estimation of air pollution exposures**

Ambient air pollutant concentrations included the 24-h average concentrations of PM_2.5_ (particulate matter with a diameter of 2.5 μm and less), PM_10_, nitrogen dioxide (NO_2_), sulfur dioxide (SO_2_), carbon monoxide (CO), and the daily maximum 8-h average concentrations of ozone (O_3_) from May 26 to July 20, 2021, were obtained from Xinxiang Environmental Monitoring Station. The distance between the participant's dwellings and the environmental monitoring site was less than 10 km. The daily average temperature and relative humidity was obtained from the China Meteorological Data Network. In addition to single-day lag models (lag0 to lag7), moving average lag models (lag01 and lag07) were also used to assess the impacts of air pollution exposure.

**2.3 Measurements of peripheral blood biomarkers**

15.0 ml peripheral venous blood was drawn by medical staff from each participant fasting for at least 8 h. 10.0 ml of blood was put into an anticoagulant-treated tube and gently mixed before cell counting. The other 5.0 ml of blood was drained into a tube without anticoagulant treatment and sat for 30-60 min at room temperature to collect serum for hs-CRP measurement. WBC, EOS, BAS, MON, LYM, and NEU were counted using an automated hematology analyzer (XS-500i, Sysmex Corporation, Kobe, Japan) and NLR calculated. Serum hs-CRP was measured using an automated clinical chemistry analyzer (CS-2000, Dirui Industrial, China). The biomarkers were examined by a certified clinical laboratory with standardized operating and quality control procedures.

**2.4 Statistical analyses**

Continuous variables' normal and non-normal distributions were presented, respectively, as mean (standard deviation) and median (interquartile range); categorical variables were expressed as number (percentage).

The association between exposure to air pollution and meteorological factors were assessed by Spearman correlation analyses. The short-term impact of ambient air pollution on blood biomarkers was evaluated using multivariable linear regression models. To assess the lag-associated effects of air pollution exposure, the model incorporated a spectrum of lagged, including single-day lags and moving averages of multiple lagged days (lag0-7, 01-07). Blood biomarker values were normalized using log10 transformation before being included into the models. Those estimated effects were displayed as a percentage change (95% CI) for every 10 μg/m^3^ increasing concentration of each air pollutant.

For each air pollutant, its effect on a single biomarker was evaluated individually. The air pollution exposure model was adjusted for age, BMI, and category-type variables including sex, villages, marital, smoking and drinking status, education, average monthly income, and physical activity levels, CCI, allergy, and meteorological factors including temperature and relative humidity. For the latter, only those lag dates that produced minimum moving average values of Akaike Information Criterion (AIC) were selected.

To investigate the confounding effects of sex (male vs. female), BMI (< 24 vs. ≥ 24 kg/m^2^), smoking status (current smoker, never or former smokers), and age (≥ 60 vs. < 60 years) on the relationship between air pollution exposure and blood biomarkers, stratification analyses were preformed. To verify the robustness of the estimated effects and allow for the adjustment of co-pollutants, sensitivity analyses were carried out with two-pollutant models.

R software (version 4.1.3) was used for all data analyses, and the "ggplot2" package was used to visualize data. The threshold for statistical significance was set at *P*<0.05.

**3. Results**

**3.1 Demographic characteristics of the participants**

Demographic characteristics and blood biomarkers of the participants are presented in **Table 1**. In this study, 2157 males (37.1%) and 3659 females (62.9%) with an average age of 57.79 years and BMI of 25.59 kg/m^2^ were recruited. Of 5816 subjects, 16.5% were current smokers and 77.6% former smokers; 63.8% had high physical activity and 31.5% moderate activity. The median counts for WBC, EOS, BAS, MON, LYM, and NEU were 5.97×10^9^/L, 0.09×10^9^/L, 0.03×10^9^/L, 0.27×10^9^/L, 1.98×10^9^/L, and 3.48×10^9^/L, respectively, and the median of NLR was 1.7%. Meanwhile, the mean serum level of hs-CRP was 2.04 mg/L.

**3.2 Correlations of ambient air pollutants and meteorological variables**

**Table 2** shows the distribution of air pollutant concentrations and meteorological variables over the survey period. The median levels of PM_2.5_, PM_10_, SO_2_, NO_2_, O_3_, and CO were 24.50 (8.0-65.0), 56.50 (20.0-148.0), 10.00 (2.0-16.0), 19.00 (7.0-37.0), 86.00 (39.6-68.0) μg/m^3^ and 0.60 (0.3-1.3) μg/m^3^, respectively. PM_2.5_ and PM_10_ concentrations were above the current World Health Organization’s air quality guidelines. In addition, the median temperature and relative humidity were 28.10 (23.3-31.7) ℃ and 64.5 (26-100) %, respectively.

The associations of air pollutant concentrations with meteorological factors were assessed with Spearman correlation analyses. The varied correlations were shown in **Fig. S1**.

**3.3 Effects of air pollution exposure on blood biomarkers**

After adjustment for confounding factors, the associations between air pollutant levels and blood cellular biomarkers are depicted in **Fig 1**.

As shown in **Fig 1**, exposure to PM_2.5_ (lag5, lag6) or CO (lag5, lag6, lag06, and lag07) was positively correlated to total WBC counts, respectively. Every 10 μg/m^3^ increment in PM_2.5_ (lag5) and CO (lag07) was related to 0.51% (95% CI: 0.19%, 0.82%) and 0.04% (95 CI: 0.01%, 0.07%) increase in total WBC counts, respectively. In contrast, SO_2_ exposure was negatively correlated with this parameter, each 10 μg/m^3^ increase in SO_2_ (lag5) was negatively associated with -1.34% (95CI: -0.08%, -2.6%) decrease in WBC counts. No correlations were detected between PM_10_, NO_2_, or O_3_ exposure with WBC count.

In addition, **Fig 1** also shows the associations of air pollution exposures with EOS counts. Exposures to PM_2.5_ (lag1, 6, 7, 03-07), PM_10_ (lag1, 3, 6, 02-07), NO_2_ (lag7), CO (lag7) and O_3_ (lag1, 6), but not for SO_2_ exposure, were negatively associated with EOS counts. The strongest effects on EOS was seen with PM_2.5_ (lag07), PM_10_ (07), NO_2_ (lag7), CO (lag7), and O_3_ (lag1) exposures. Each 10 μg/m^3^ rise in PM_2.5_, PM_10_, NO_2_, CO, and O_3_ levels under these exposure scenarios was related to a decrease of -2.99% (95 CI: -0.99%, -5%), -1.08% (95 CI: -0.29%, -1.88%), -2.48% (95 CI: -0.85%, -4.12%), -0.07% (95 CI: -0.01%, -0.12%) and -0.38% (95 CI: -0.04%, -0.73%) in EOS, respectively.

The negative effects of air pollution on BAS were also detected as for EOS. Each 10 μg/m^3^ increment in PM_2.5_, SO_2,_ and CO concentrations was related to a decline of -0.65% (95% CI: 0.00%, -1.30%), -2.22% (95% CI: -0.03%, -4.40%), and -0.04% (95% CI: 0.00%, -0.08%) in BAS (lag1), respectively. The decreasing trends for BAS count following PM_10_ and NO_2_ exposure were observed, but not significant.

Short-term exposure to SO_2_, NO_2,_ or O_3_ posed negative effect on MON count. Each 10 μg/m^3^ increase in SO_2_ (lag5), NO_2_ (lag7), and O_3_ (lag04) was associated with a decrease of -1.61% (95% CI: -0.28%, -2.94%), -0.89% (95% CI: -0.27%, -1.51%) and -0.31% (95% CI: 0.08%, -0.54%) in MON count, respectively. Nevertheless, there was no significant associations between PM_2.5_, PM_10_, or CO levels and MON count.

The concentrations of PM_2.5_ (lag1, 7), PM_10_ (lag1-3, lag6 and lag01-07), SO_2_ (lag7, lag07) and NO_2_ (lag7) were negatively associated with LYM count, while CO (lag0, 5) and O_3_ (lag0, 4) were positively related to this parameter. And the largest estimated effects on LYM for the aforementioned air pollutants were observed with PM_2.5_ (lag1) (-0.39% CI: -0.02, -0.76), PM_10_ (lag07) (-0.59%, 95% CI: -0.23%, -0.95%), SO_2_ (lag07) (-5.54%, 95% CI: -1.53%, -9.56%), NO_2_ (lag7) (-0.90%, 95% CI: -0.14%, -1.66 %), CO (lag0) (0.03%, 95% CI: 0.01%, 0.05%), or O_3_ (lag4) (0.15%, 95% CI: 0.04%, 0.27%).

There existed significantly positive associations of PM_2.5_ (lag5-7, lag06-07), PM_10_ (lag7), SO_2_ (lag7), or CO (lag2,5-7, lag 05-07) exposure with NEU count, respectively. The maximum effects of air pollution exposure on NEU count occurred with PM_2.5_ (lag07), PM_10_ (lag7), SO_2_ (lag7), or CO (lag07). Under this circumstance, each 10 ug/m^3^ increase in PM_2.5_, PM_10_, SO_2_, and CO correlated to an increase of 1.23% (95%CI: 0.37%, 2.09%), 0.17% (95%CI: 0.00%, 0.33%), 2.51% (95%CI: 0.48%, 4.54%), and 0.06% (95%CI: 0.03%, 0.1%) in NEU count.

As illustrated in **Fig 2**, significantly positive associations were revealed between PM_2.5_ (lag1, 5-7, lag05-07), PM_10_ (lag1, 5, 6, lag 02, 05-07), SO_2_ (lag6, lag7), or CO (lag6, lag7, lag07) exposure and NLR. Moreover, PM_2.5_ (lag07), PM_10_ (lag07), SO_2_ (lag7), and CO (lag07) posed strongest effects, corresponding to rises in NLR of 1.82% (95%CI: 0.83%, 2.82%), 0.57% (95%CI: 0.17%, 0.97%), 4.99% (95%CI: 2.62%, 7.36%), and 0.05% (95%CI: 0.01%, 0.1%).

Short-term air pollution exposure was found to be significantly related to serum hs-CRP **(Fig 2)**. Specifically, PM_2.5_ (lag5-7, lag02-07), PM_10_ (lag4-7, lag06, lag07), SO_2_ (lag1-3, lag01-07), NO_2_ (lag7), CO (lag2-7, lag01-07), and O_3_ (lag1-7, lag01-07) exposures had significantly positive associations with serum levels of hs-CRP. Hs-CRP increased by 4.51% (95%CI: 2.72%, 6.29%), 1.21% (95%CI: 0.45%, 1.97%), 18.96% (95%CI: 9.66%, 28.26%), 2.08% (95%CI: 0.83%, 3.33%), 0.17% (95%CI: 0.1%, 0.24%), and 1.54% (95%CI: 1.04%, 2.05%) for each 10 μg/m^3^ increase in PM_2.5_ (lag07), PM_10_ (lag07), SO_2_ (lag07), NO_2_ (lag7), CO (lag07), and O_3_ (lag07), respectively

**3.4 Confounding factor stratification and two-pollutant model analyses**

The stratification by sex, BMI, smoking status, and age was performed. It was shown that the impacts of PM_2.5_ and SO_2_ on EOS were significantly higher in males than in females, but no sex difference in NLR (**Fig. 3**). The effects of SO_2_ on NEU and hs-CRP were significantly greater in people with a BMI less than 24 kg/m^2^ than people with a BMI ≥24 kg/m^2^. This was not seen for PM_2.5_ and PM_10_ in those with a BMI less than 24 kg/m^2^ (**Fig. 4**). Current smoking was found to strengthen SO_2_-induced decrease in EOS counts than non-smoking. Additionally, the effects of PM_10_ exposure on NLR and NEU were significantly stronger in smokers than in non-smokers (**Fig. 5**). Older ages (≥ 60 years) significantly increased the effects of PM_2.5_ exposure on EOS and LYM as well as the effect of SO_2_ on LYM than younger ages (< 60 years) (**Fig. 6**).

In the two-pollutant model analyses, significant associations were detected between PM_2.5_ and counts of WBC, NEU, and NLR, as well as serum levels of hs-CRP after controlling other co-pollutants. In addition, although the significant association of PM_2.5_ with EOS or BAS became insignificant after inclusion of PM_10_ or SO_2_ in the two-pollutant model, these resultant differences were not significant (**Table 3**). Given minimal differences in the impacts of air pollution between one-pollutant and two-pollutant models, it was assumed that the influence of each air pollutant on blood biomarkers was predominant (**Table S1-5**).

**4. Discussion**

Thus far, the evidence of air pollution exposure in rural areas on peripheral white blood cells and inflammation biomarkers is scarce and inconsistent. This study revealed significant associations of short-term exposure to ambient air pollution with peripheral white blood cells and a serum inflammatory biomarker of the population living in rural areas of Northern Henan Province, China. Interestingly, men, normal BMI, current smokers, and old age rendered the participants more susceptible to the harmful effects induced by air pollution exposure.

WBC are considered as systemic effector cells of inflammatory stimuli [25]. In the present study, we found that exposure to PM_2.5_ and CO significantly increased WBC count, while SO_2_ exposure markedly reduced WBC count. These findings were consistent with the previous reports. For example, previous studies with an American population and a Chinese community have shown that short-term exposure to ambient PM_2.5_ enhances counts of circulating WBC [26, 27]. Increased WBC count following CO exposure was also reported in a Japanese population [28]. The underlying mechanism for WBC effect is assumed to be due to released cytokines in the circulation, further rendering leukocyte mobilization from bone marrow [29]. An interesting finding from 125 healthy young adults was that exposure to lower levels of gaseous and particulate pollution did not significantly boost WBC counts [30]. Another study showed that circulating WBC count was even reduced following short-term exposure to atmospheric PM in postmenopausal women aged 50–79 years [31]. Whether these discrepancies in WBC effect were due to the curvilinear association of SO_2_ with WBC counts remains unclear [32]. In addition, the variations in the study subjects' demographics and exposure to air pollution over time may also have an impact.

The present study discovered that the subtypes of WBC including EOS, BAS, and MON were also significantly associated with air pollution exposure. Specifically, exposure to PM_2.5_, PM_10_, NO_2_, CO, and O_3_ were negatively related to EOS counts, while exposure to PM_2.5_, SO_2_, and CO were also negatively related to BAS counts. Moreover, gaseous air pollutants including SO_2_, NO_2_, and O_3_ exposures were negatively associated with MON counts. In addition, this study also showed that short-term exposures to PM_2.5_, PM_10_, SO_2_, and NO_2_ decreased LYM counts but CO and O_3_ exposures increased LYM counts. Noteworthy, exposures to PM_2.5_, PM_10_, SO_2_, and CO were significantly correlated with the increase in NEU counts. These findings were in line with some previous studies. For instance, in a study with 31 subjects from five different locations, Steenhof et al. reported a negative correlation between NO_2_ exposure and LYM counts, and a positive association of PM with NEU counts [15]. In another study of healthy men in North Carolina, USA, Riediker et al. showed that exposure to PM_2.5_ was negatively correlated with LYM and positively associated with NEU count [33]. Additionally, a Copenhagen research showed that short-term exposure to ambient CO increased counts of NEU and LYM [28]. However, there was one study showing no correlation between exposure to air pollution and BAS, EOS, and MOS counts of healthy people's [34]. Another study with non-smoking, healthy adults living in northern France demonstrated that exposure to lower concentrations of O_3_ was associated with a significantly higher EOS count [35]. In addition, an investigation of 15 healthy volunteers showed a decrease in LYM after short-term exposure to O_3_ [36]. A recent study with an urban population reported a significant positive association between NEU and LYM counts and short-term exposure to air pollutants (PM_2.5_, PM_10_, SO_2_, NO_2_, CO, O_3_) [13]. Overall, the associations of ambient air pollution with the subtypes of WBC counts appeared to be air pollutant- and cell type-dependent, and were also affected by the participant’s health status, air pollution levels, and the physicochemical characteristics of air pollutants.

NLR has been proposed as a more sensitive and systemic biomarker of inflammation and immune responses induced by diverse stimuli [37–40]. However, the studies used NLR as a biomarker of air pollutant effects on humans are limited and inconsistent. For instance, a Swedish epidemiological investigation revealed that moderate PM_2.5_ and PM_10_ exposures were associated with increased NLR [41]. In contrast, a study of Chinese schoolchildren did not find significant alteration in NLR between the polluted and control areas [42]. Interestingly, according to a cross-sectional study of urban Chinese adults, exposure to CO was positively correlated with NLR whereas exposure to PM10 negatively correlated with NLR[13]. This present study in rural areas showed an association between short-term exposures to PM_2.5_, PM_10_, SO_2_, and CO with elevations in NLR, suggesting that exposure to air pollutants induced systemic immunological and inflammatory responses.

Hs-CRP is the specific biomarker of low but persistent levels of inflammation [20]. A previous study has demonstrated that short-term exposure to either PM_2.5_ or PM_10_ raised hs-CRP levels, with PM_2.5_ having a larger effect than PM_10_ [43]. In addition, gaseous air pollutants including SO_2_, NO_2_, and CO also induced significant increases in hs-CRP [19]. In line with these observations, this study revealed positive correlations between PM_2.5_, PM_10_, SO_2_, NO_2_, CO, or O_3_ and hs-CRP, and PM_2.5_ also showed a stronger effect than PM_10_. Whether air pollution-induced oxidative stress is involved in this process remains to be elucidated [44, 45].

This study revealed that men, individuals with normal BMI, current smokers, and those older than 60 years were also shown to be more vulnerable to the harmful effects of acute exposure to air pollutants. The impact of sex factor may be due to biological and lifestyle differences between males and females, the latter having better innate immunity than men [46, 47]. The effects of SO_2_ exposure on NEU and hs-CRP were more evident in the non-overweight population than in the overweight population in this study. Similarly, a previous research demonstrated that the effect of PM_10_ on hs-CRP was more evident in non-obese individuals [48]. The explanation for this observation could be that the people with obesity or overweight had slightly higher baseline of oxidative stress and inflammation, rendering the effects of air pollution in these people difficult to identify [49]. Both air pollutants and cigarettes have been reported to induce acute epithelial injury and neutrophil inflammation [50, 51]. It is assumed that the airways of smokers are vulnerable to air pollutant adverse effects [52–54]. With regard to the age, the elderly are more susceptible to health problems when exposed to air pollutants given their age-related impairment of immune system, ineffective cardiovascular and respiratory systems, and existence of other adverse co-factors [55].

The present study was conducted on a rural population to assess the effects of short-term exposure to air pollution on peripheral white blood cells and serum inflammatory biomarker. A study conducted on a population in Kaohsiung City, Japan, showed similar results, with air pollution exposure being associated with white blood cells alterations, implying that air pollution is associated with systemic inflammation in humans [28]. Another study conducted on 22 healthy young subjects from highly polluted urban areas and low-polluted rural areas, respectively, demonstrated that air pollution was associated with hs-CRP and white blood cells levels, with a greater effect on leukocyte counts in urban populations probably due to differences in air pollution levels, sources and other socioeconomic factors between urban and rural areas [56].

This study used peripheral leukocytes and a serum inflammatory biomarker as exposure outcomes to assess the potential response to short-term air pollution exposure on populations in rural areas, with the innovative use of NLR as a composite marker. The strength of this study mainly lied on the use of comprehensive data of sociodemographic characteristics, lifestyle, health conditions, and other factors. In addition, all results were adjusted for potential confounders, which made the findings of the study more robust and credible. Several limitations should also be pointed out for this study. First, air pollution exposure might be underestimated using monitoring station data since environmental monitoring stations might not accurately capture personal exposure levels, especially if individuals spend significant time indoors or away from the monitoring locations. In addition, air pollutant levels could vary significantly over small distances and short time periods, which might not be captured fully by the data from monitoring stations. Second, there could be additional uncontrolled confounding factors in the study, such as indoor air pollution or occupational exposures, or socioeconomic status, which might influence both exposure and biomarker levels. Third, some biomarkers used in this study might not be sensitive enough to detect subtle changes caused by short-term exposures, or they may not reflect longer-term health effects.

**5. Conclusions**

This cross-sectional study on a rural population revealed that short-term exposure to ambient air pollutants was associated with alterations of blood inflammatory biomarker, offering new epidemiological evidence for understanding air pollution adverse effects on the health of rural residents. Examination of blood samples for inflammatory biomarkers is important in these rural populations because it not only helps individuals and communities understand their health status, but also helps the early detection of many common diseases, such as diabetes and hypertension. This may reduce healthcare costs and improve the overall health of the rural population. Future researches will investigate long-term exposure to air pollution on peripheral white blood cells and inflammation biomarkers to test the validity of these cellular and molecular biomarkers in rural populations. Animal models will also be introduced to examine the causal relationship between exposure to ambient air pollutants collected from rural areas and systemic inflammation, and examine intervention measures.

**References**

1. Schraufnagel DE, Balmes JR, Cowl CT, De Matteis S, Jung S-H, Mortimer K, et al. Air Pollution and Noncommunicable Diseases: A Review by the Forum of International Respiratory Societies’ Environmental Committee, Part 2: Air Pollution and Organ Systems. Chest. 2019;155:417–26.

2. Liu Y, Pan J, Zhang H, Shi C, Li G, Peng Z, et al. Short-Term Exposure to Ambient Air Pollution and Asthma Mortality. Am J Respir Crit Care Med. 2019;200:24–32.

3. Cohen AJ, Brauer M, Burnett R, Anderson HR, Frostad J, Estep K, et al. Estimates and 25-year trends of the global burden of disease attributable to ambient air pollution: an analysis of data from the Global Burden of Diseases Study 2015. Lancet Lond Engl. 2017;389:1907–18.

4. Liu C, Chen R, Sera F, Vicedo-Cabrera AM, Guo Y, Tong S, et al. Ambient Particulate Air Pollution and Daily Mortality in 652 Cities. N Engl J Med. 2019;381:705–15.

5. Pope CA, Bhatnagar A, McCracken JP, Abplanalp W, Conklin DJ, O’Toole T. Exposure to Fine Particulate Air Pollution Is Associated With Endothelial Injury and Systemic Inflammation. Circ Res. 2016;119:1204–14.

6. Li Z, Liu Q, Xu Z, Guo X, Wu S. Association between short-term exposure to ambient particulate air pollution and biomarkers of oxidative stress: A meta-analysis. Environ Res. 2020;191:110105.

7. Tang H, Cheng Z, Li N, Mao S, Ma R, He H, et al. The short- and long-term associations of particulate matter with inflammation and blood coagulation markers: A meta-analysis. Environ Pollut Barking Essex 1987. 2020;267:115630.

8. Salvi S, Blomberg A, Rudell B, Kelly F, Sandström T, Holgate ST, et al. Acute inflammatory responses in the airways and peripheral blood after short-term exposure to diesel exhaust in healthy human volunteers. Am J Respir Crit Care Med. 1999;159:702–9.

9. Mumby S, Chung KF, Adcock IM. Transcriptional Effects of Ozone and Impact on Airway Inflammation. Front Immunol. 2019;10:1610.

10. Zhang JJ, Wei Y, Fang Z. Ozone Pollution: A Major Health Hazard Worldwide. Front Immunol. 2019;10:2518.

11. Chen J-C, Schwartz J. Metabolic Syndrome and Inflammatory Responses to Long-Term Particulate Air Pollutants. Environ Health Perspect. 2008;116:612–7.

12. Bekwelem W, Lutsey PL, Loehr LR, Agarwal SK, Astor BC, Guild C, et al. White Blood Cell Count, C-Reactive Protein, and Incident Heart Failure in the Atherosclerosis Risk in Communities (ARIC) Study. Ann Epidemiol. 2011;21:739–48.

13. Qiu W, He H, Wang B, Wang D, Mu G, Xu T, et al. Short-term impacts of air pollution on the platelet-lymphocyte ratio and neutrophil-lymphocyte ratio among urban adults in China. J Environ Sci China. 2023;125:101–11.

14. Hung S-C, Cheng H-Y, Yang C-C, Lin C-I, Ho C-K, Lee W-H, et al. The Association of White Blood Cells and Air Pollutants-A Population-Based Study. Int J Environ Res Public Health. 2021;18:2370.

15. Steenhof M, Janssen NAH, Strak M, Hoek G, Gosens I, Mudway IS, et al. Air pollution exposure affects circulating white blood cell counts in healthy subjects: the role of particle composition, oxidative potential and gaseous pollutants - the RAPTES project. Inhal Toxicol. 2014;26:141–65.

16. Buonacera A, Stancanelli B, Colaci M, Malatino L. Neutrophil to Lymphocyte Ratio: An Emerging Marker of the Relationships between the Immune System and Diseases. Int J Mol Sci. 2022;23:3636.

17. Zahorec R. Neutrophil-to-lymphocyte ratio, past, present and future perspectives. Bratisl Lek Listy. 2021;122:474–88.

18. Sproston NR, Ashworth JJ. Role of C-Reactive Protein at Sites of Inflammation and Infection. Front Immunol. 2018;9:754.

19. Xu Z, Wang W, Liu Q, Li Z, Lei L, Ren L, et al. Association between gaseous air pollutants and biomarkers of systemic inflammation: A systematic review and meta-analysis. Environ Pollut Barking Essex 1987. 2022;292 Pt A:118336.

20. Darras-Hostens M, Achour D, Muntaner M, Grare C, Zarcone G, Garçon G, et al. Short-term and residential exposure to air pollution: Associations with inflammatory biomarker levels in adults living in northern France. Sci Total Environ. 2022;833:154985.

21. Song J, Zhu J, Tian G, Li H, Li H, An Z, et al. Short time exposure to ambient ozone and associated cardiovascular effects: A panel study of healthy young adults. Environ Int. 2020;137:105579.

22. Song J, Liu Y, Lu M, An Z, Lu J, Chao L, et al. Short-term exposure to nitrogen dioxide pollution and the risk of eye and adnexa diseases in Xinxiang, China. Atmos Environ. 2019;218:117001.

23. Hou J, Liu X, Tu R, Dong X, Zhai Z, Mao Z, et al. Long-term exposure to ambient air pollution attenuated the association of physical activity with metabolic syndrome in rural Chinese adults: A cross-sectional study. Environ Int. 2020. https://doi.org/10.1016/j.envint.2020.105459.

24. Charlson ME, Pompei P, Ales KL, MacKenzie CR. CCI: A new method of classifying prognostic comorbidity in longitudinal studies: development and validation. J Chronic Dis. 1987;40:373–83.

25. Lee H, Myung W, Jeong B-H, Choi H, Jhun BW, Kim H. Short- and long-term exposure to ambient air pollution and circulating biomarkers of inflammation in non-smokers: A hospital-based cohort study in South Korea. Environ Int. 2018;119:264–73.

26. Wei T, Chen C, Yang Y, Li L, Wang J, Ye M, et al. Associations between short-term exposure to ambient air pollution and lung function in adults. J Expo Sci Environ Epidemiol. 2023. https://doi.org/10.1038/s41370-023-00550-0.

27. Schwartz J. Air pollution and blood markers of cardiovascular risk. Environ Health Perspect. 2001;109 Suppl 3 Suppl 3:405–9.

28. Hung S-C, Cheng H-Y, Yang C-C, Lin C-I, Ho C-K, Lee W-H, et al. The Association of White Blood Cells and Air Pollutants-A Population-Based Study. Int J Environ Res Public Health. 2021;18:2370.

29. Khafaie MA, Salvi SS, Ojha A, Khafaie B, Gore SS, Yajnik CS. Systemic inflammation (C-reactive protein) in type 2 diabetic patients is associated with ambient air pollution in Pune City, India. Diabetes Care. 2013;36:625–30.

30. Rich DQ, Kipen HM, Huang W, Wang G, Wang Y, Zhu P, et al. Association between changes in air pollution levels during the Beijing Olympics and biomarkers of inflammation and thrombosis in healthy young adults. JAMA. 2012;307:2068–78.

31. Gondalia R, Holliday KM, Baldassari A, Justice AE, Stewart JD, Liao D, et al. Leukocyte Traits and Exposure to Ambient Particulate Matter Air Pollution in the Women’s Health Initiative and Atherosclerosis Risk in Communities Study. Environ Health Perspect. 2020;128:17004.

32. Liao D, Heiss G, Chinchilli VM, Duan Y, Folsom AR, Lin HM, et al. Association of criteria pollutants with plasma hemostatic/inflammatory markers: A population-based study. J Expo Anal Environ Epidemiol. 2005;15:319–28.

33. Riediker M, Cascio WE, Griggs TR, Herbst MC, Bromberg PA, Neas L, et al. Particulate matter exposure in cars is associated with cardiovascular effects in healthy young men. Am J Respir Crit Care Med. 2004;169:934–40.

34. Zuurbier M, Hoek G, Oldenwening M, Meliefste K, Krop E, van den Hazel P, et al. In-traffic air pollution exposure and CC16, blood coagulation, and inflammation markers in healthy adults. Environ Health Perspect. 2011;119:1384–9.

35. Dauchet L, Hulo S, Cherot-Kornobis N, Matran R, Amouyel P, Edmé J-L, et al. Short-term exposure to air pollution: Associations with lung function and inflammatory markers in non-smoking, healthy adults. Environ Int. 2018;121:610–9.

36. Stiegel MA, Pleil JD, Sobus JR, Madden MC. Inflammatory Cytokines and White Blood Cell Counts Response to Environmental Levels of Diesel Exhaust and Ozone Inhalation Exposures. PloS One. 2016;11:e0152458.

37. Shi G, Zhao J-W, Ming L. Clinical significance of peripheral blood neutrophil-lymphocyte ratio and platelet- lymphocyte ratio in patients with asthma. Nan Fang Yi Ke Da Xue Xue Bao. 2017;37:84–8.

38. Prats-Puig A, Gispert-Saüch M, Díaz-Roldán F, Carreras-Badosa G, Osiniri I, Planella-Colomer M, et al. Neutrophil-to-lymphocyte ratio: an inflammation marker related to cardiovascular risk in children. Thromb Haemost. 2015;114:727–34.

39. Aksoy E, Karakurt Z, Gungor S, Ocakli B, Ozmen İ, Yildirim E, et al. Neutrophil to lymphocyte ratio is a better indicator of COPD exacerbation severity in neutrophilic endotypes than eosinophilic endotypes. Int J Chron Obstruct Pulmon Dis. 2018;13:2721–30.

40. Afari ME, Bhat T. Neutrophil to lymphocyte ratio (NLR) and cardiovascular diseases: an update. Expert Rev Cardiovasc Ther. 2016;14:573–7.

41. Azzouz M, Xu Y, Barregard L, Fagerberg B, Zöller B, Molnár P, et al. Air pollution and biomarkers of cardiovascular disease and inflammation in the Malmö Diet and Cancer cohort. Environ Health Glob Access Sci Source. 2022;21:39.

42. Li X, Zhang X, Zhang Z, Han L, Gong D, Li J, et al. AP 免疫和系统炎症标志物 中国儿童 Air pollution exposure and immunological and systemic inflammatory alterations among schoolchildren in China. Sci Total Environ. 2019;657:1304–10.

43. Liu Q, Gu X, Deng F, Mu L, Baccarelli AA, Guo X, et al. Ambient particulate air pollution and circulating C-reactive protein level: A systematic review and meta-analysis. Int J Hyg Environ Health. 2019;222:756–64.

44. Yan Z, Jin Y, An Z, Liu Y, Samet JM, Wu W. Inflammatory cell signaling following exposures to particulate matter and ozone. Biochim Biophys Acta. 2016;1860:2826–34.

45. Chen R, Hu B, Liu Y, Xu J, Yang G, Xu D, et al. Beyond PM2.5: The role of ultrafine particles on adverse health effects of air pollution. Biochim Biophys Acta. 2016;1860:2844–55.

46. Ghazeeri G, Abdullah L, Abbas O. Immunological differences in women compared with men: overview and contributing factors. Am J Reprod Immunol N Y N 1989. 2011;66:163–9.

47. Chen W, Mempel M, Schober W, Behrendt H, Ring J. Gender difference, sex hormones, and immediate type hypersensitivity reactions. Allergy. 2008;63:1418–27.

48. Pilz V, Wolf K, Breitner S, Rückerl R, Koenig W, Rathmann W, et al. C-reactive protein (CRP) and long-term air pollution with a focus on ultrafine particles. Int J Hyg Environ Health. 2018;221:510–8.

49. Gutiérrez-Solis AL, Garrido-Dzib AG, Rochel-Pérez A, Magallón-Zertuche V, Chávez-Loría G, Medina-Vera I, et al. Oxidative Stress Biomarkers in Mexican Subjects with Overweight and Obesity: A Systematic Review. Metab Syndr Relat Disord. 2023;21:188–96.

50. Pouwels SD, Zijlstra GJ, van der Toorn M, Hesse L, Gras R, Ten Hacken NHT, et al. Cigarette smoke-induced necroptosis and DAMP release trigger neutrophilic airway inflammation in mice. Am J Physiol Lung Cell Mol Physiol. 2016;310:L377-386.

51. Wigenstam E, Elfsmark L, Bucht A, Jonasson S. Inhaled sulfur dioxide causes pulmonary and systemic inflammation leading to fibrotic respiratory disease in a rat model of chemical-induced lung injury. Toxicology. 2016;368–369:28–36.

52. Danov O, Wolff M, Bartel S, Böhlen S, Obernolte H, Wronski S, et al. Cigarette Smoke Affects Dendritic Cell Populations, Epithelial Barrier Function, and the Immune Response to Viral Infection With H1N1. Front Med. 2020;7:571003.

53. Heijink IH, Brandenburg SM, Postma DS, van Oosterhout AJM. Cigarette smoke impairs airway epithelial barrier function and cell-cell contact recovery. Eur Respir J. 2012;39:419–28.

54. Mehta H, Nazzal K, Sadikot RT. Cigarette smoking and innate immunity. Inflamm Res Off J Eur Histamine Res Soc Al. 2008;57:497–503.

55. Arbex MA, Santos U de P, Martins LC, Saldiva PHN, Pereira LAA, Braga ALF. Air pollution and the respiratory system. J Bras Pneumol Publicacao Of Soc Bras Pneumol E Tisilogia. 2012;38:643–55.

56. Jaafari J, Naddafi K, Yunesian M, Nabizadeh R, Hassanvand MS, Shamsipour M, et al. Associations between short term exposure to ambient particulate matter from dust storm and anthropogenic sources and inflammatory biomarkers in healthy young adults. Sci Total Environ. 2021;761:144503.

**Acknowledgments**

The field investigator and all participants are acknowledged by the authors for their contributions to this study.

**Declarations**

**Ethics approval and consent to participate**

The Ethics Committee of Xinxiang Medical University for Human Studies authorized this investigation (XYLL-2016242). Informed consent was obtained from all individual participants included in the study.

**Consent for publication**

Not applicable.

**Availability of data and materials**

The data used and/or analyzed during the study will be available upon the request.

**Competing interests**

The authors declare that they have no known competing financial interests or personal relationships that influence the work reported in this paper.

**Funding**

This work was supported by the National Key Research and Development Program Precision Medicine Initiative of China (2016YFC0900803), and the National Natural Science Foundation of China (81961128031; 81573112).

**Author’s contributions**

Yishu Yang: Investigation, Methodology, Data collection, Verification, Visualization, Software, Formal analysis, writing – original draft. Hui Wu: Methodology, Software, Validation, Visualization, Writing-review. Fei Xu: Investigation, Methodology, Validation. Shuaiqi Zhao: Methodology, Validation. Yuling Zeng: Methodology, Validation. Ling Zhang: Methodology, Validation. Zhen An: Investigation, Methodology, Validation. Huijun Li: Investigation, Methodology, Validation. Juan Li: Investigation, Methodology, Validation. Jie Song: Methodology, Software, Validation, Visualization. Weidong Wu: Conceptualization, Funding acquisition, Supervision, Writing-review & editing, Project administration, Resources, Validation. All authors read and approved the final manuscript.

**Figure legends**

**Figure 1.** **Association of short-term exposure to air pollutants with WBC and subtypes at different lags.** The effects were presented as percentage changes (%) per 10 μg/m^3^ changes in air pollutant concentrations. Abbreviations: WBC, white blood cells; EOS, eosinophils; BAS, basophils; MON, monocytes; LYM, lymphocytes; NEU, neutrophils.

**Figure 2.** **Association of short-term exposure to air pollutants with NLR and hs-CRP at different lags**. The effects were represented as percentage changes (%) for each 10 μg/m^3^ changes in air pollutants concentration. Abbreviations: NLR, neutrophil-lymphocyte ratio; hs-CRP, high-sensitivity C-reactive protein.

**Figure 3.** **Changes in age-stratified effects on blood biomarkers associated with a 10 μg/m^3^ increase in concentrations of air pollutants**. The effects were represented as percentage changes (%) for each 10 μg/m^3^ changes in air pollution concentrations. Older, age≥ 60 years; Younger, age< 60 years.

**Figure 4. Changes in sex-stratified effects on blood biomarkers associated with a 10 μg/m^3^ increase in concentrations of air pollutants**. The effects were represented as percentage changes (%) for every 10 μg/m^3^ changes in air pollution concentrations.

**Figure 5.** **Changes in BMI-stratified effects on blood biomarkers associated with a 10 μg/m^3^ increase in concentrations of air pollutants**. The effects were represented as percentage changes (%) for every 10 μg/m^3^ changes in air pollution concentrations. Normal, BMI < 24 kg/m^2^; Overweight, BMI ≥ 24 kg/m^2^.

**Figure 6.** **Changes in smoking-stratified effects on blood biomarkers associated with a 10 μg/m^3^ increase in concentrations of air pollutants**. The effects were represented as percentage changes (%) for every 10 μg/m^3^ changes in air pollution concentrations. Non-smokers, never or former smokers; Smokers, current smokers.

**Table legends**

**Table 1:** Descriptive characteristics and blood biomarkers of the participants.

**Table 2:** Summary statistics for air pollutant concentrations and meteorological variables during the study period.

**Table 3:** Percent Changes (95% CI) of immunological and inflammatory biomarkers with a 10 μg/m^3^ increase in PM_2.5_ in two-pollutant model.

| **Table 1: Descriptive characteristics** **and blood biomarkers** **of the participants.** | | | |  |  |  |
| --- | --- | --- | --- | --- | --- | --- |
| Characteristics | | Total (5816) | |  |  |  |
| Age, years (SD) | | 57.79 (12.62) | |  |  |  |
| BMI (SD) | | 25.59 (3.63) | |  |  |  |
| Sex, n (%) | |  | |  |  |  |
| Male | | 2157 (37.1) | |  |  |  |
| Female | | 3659 (62.9) | |  |  |  |
| Villages, n (%) | |  | |  |  |  |
| Lang Gongmiao | | 3097 (53.2) | |  |  |  |
| Qi Liying | | 2608 (44.8) | |  |  |  |
| Others | | 111 (2.0) | |  |  |  |
| Marital status, n (%) | |  | |  |  |  |
| Married/cohabiting | | 5169 (88.9) | |  |  |  |
| Unmarried/separated/divorced | | 647 (11.1) | |  |  |  |
| Educational levels, n (%) | |  | |  |  |  |
| Elementary school or below | | 2050 (35.2) | |  |  |  |
| Middle school | | 2277 (39.2) | |  |  |  |
| High school or above | | 1489 (25.6) | |  |  |  |
| Average monthly income, n (%) | |  | |  |  |  |
| ＜500 RMB | | 1509 (25.9) | |  |  |  |
| 500-900 RMB | | 1734 (29.8) | |  |  |  |
| ＞500 RMB | | 2573 (44.2) | |  |  |  |
| Smoking, n (%) | |  | |  |  |  |
| Never | | 4515 (77.6) | |  |  |  |
| Current | | 960 (16.5) | |  |  |  |
| Former | | 341 (5.9) | |  |  |  |
| Drinking, n (%) | |  | |  |  |  |
| Never | | 4681 (80.5) | |  |  |  |
| Current | | 914 (15.7) | |  |  |  |
| Former | | 221 (3.8) | |  |  |  |
| Physical activity, n (%) | |  | |  |  |  |
| Low | | 276 (4.7) | |  |  |  |
| Moderate | | 3709 (63.8) | |  |  |  |
| High | | 1831 (31.5) | |  |  |  |
| CCI, n (%) | |  | |  |  |  |
| 0-level | | 2476 (42.6) | |  |  |  |
| 1-level | | 1705 (29.3) | |  |  |  |
| 2-level | | 900 (15.5) | |  |  |  |
| 3-level | | 735 (12.6) | |  |  |  |
| Allergy, n (%) | |  | |  |  |  |
| Yes | | 776 (13.3) | |  |  |  |
| No | | 5040(86.7) | |  |  |  |
| Total WBC (×10^9^/L), median (IQR) | | 5.97 (2.05) | |  |  |  |
| EOS (×10^9^/L), median (IQR) | | 0.09 (0.09) | |  |  |  |
| BAS (×10^9^/L), median (IQR) | | 0.03 (0.03) | |  |  |  |
| MON (×10^9^/L), median (IQR) | | 0.27 (0.10) | |  |  |  |
| LYM (×10^9^/L), median (IQR) | | 1.98 (0.77) | |  |  |  |
| NEU (×10^9^/L), median (IQR) | | 3.48 (1.53) | |  |  |  |
| NLR (%), median (IQR) | | 1.74 (0.88) | |  |  |  |
| hs-CRP (mg/L), median (IQR) | | 2.04 (1.92) | |  |  |  |
| Abbreviations: WBC, white blood cells; EOS, eosinophils; BAS, basophils; MON, monocytes; LYM, lymphocytes; and neutrophils (NEU); hs-CRP, high-sensitivity C-reactive protein; NLR, neutrophil-lymphocyte ratio; SD, standard deviation; IQR, interquartile. | | | |  |  |  |
| **Table 2: Summary statistics for air pollutant concentrations and meteorological variables during the study period.** | | | | | | |
| Variable | Minimum | Percentile | | | | Maximum |
|  |  | 25th | 50th | | 75th |  |
| PM_2.5_ (ug/m³) | 8.00 | 17.75 | 24.50 | | 35.25 | 65.00 |
| PM_10_ (ug/m³) | 20.00 | 46.00 | 56.50 | | 95.50 | 148.00 |
| SO_2_ (ug/m³) | 2.00 | 9.00 | 10.00 | | 12.00 | 16.00 |
| NO_2_ (ug/m³) | 7.00 | 13.00 | 19.00 | | 25.00 | 37.00 |
| CO (mg/m³) | 0.30 | 0.40 | 0.60 | | 0.70 | 1.30 |
| O_3_ (ug/m³) | 39.00 | 66.00 | 86.00 | | 106.00 | 168.00 |
| T (℃) | 23.20 | 26.03 | 28.10 | | 29.18 | 31.70 |
| RH (%) | 26.00 | 50.50 | 64.50 | | 79.50 | 100.00 |

**Fig. 1**

**
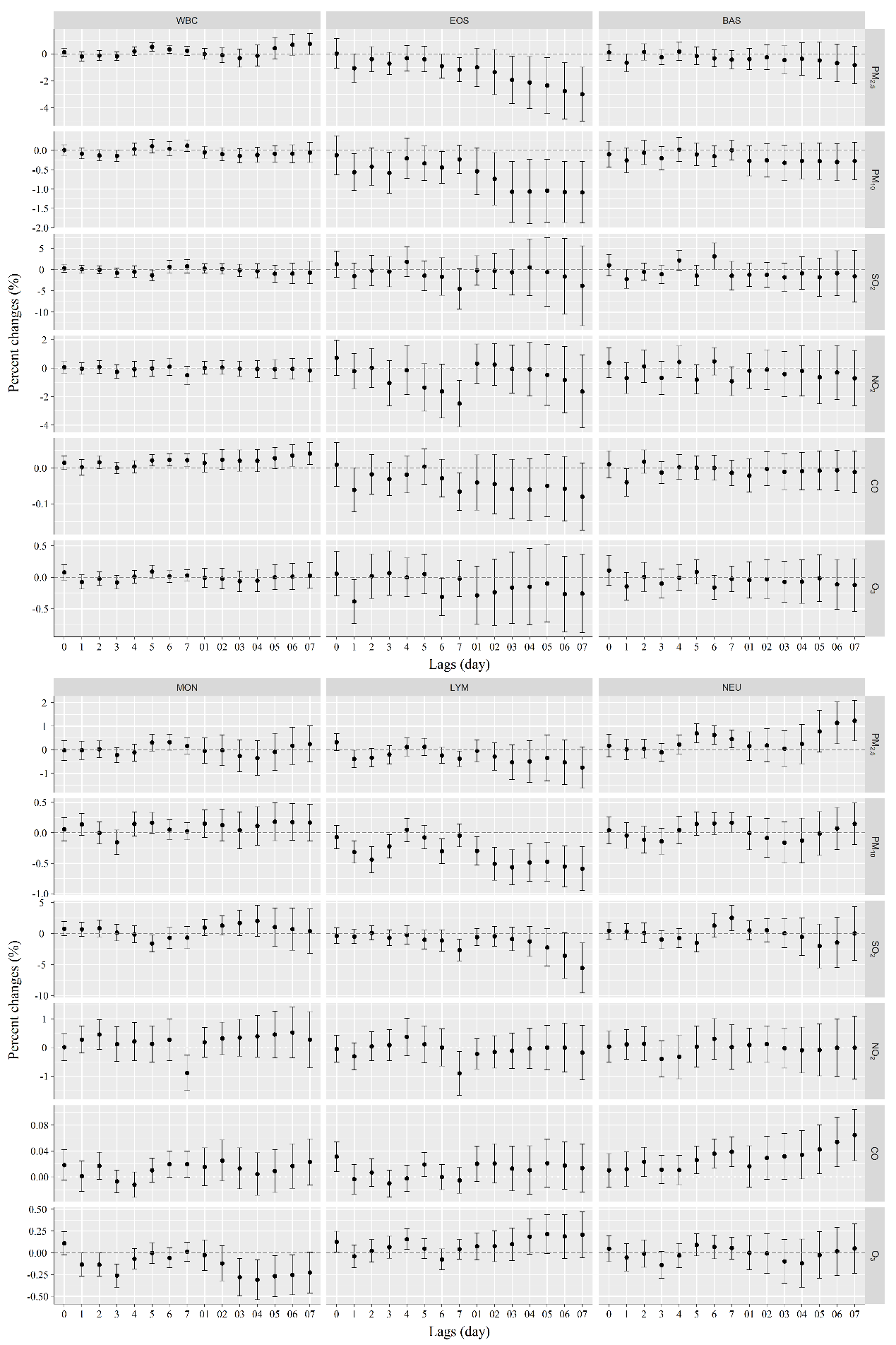
**

**Fig. 2**


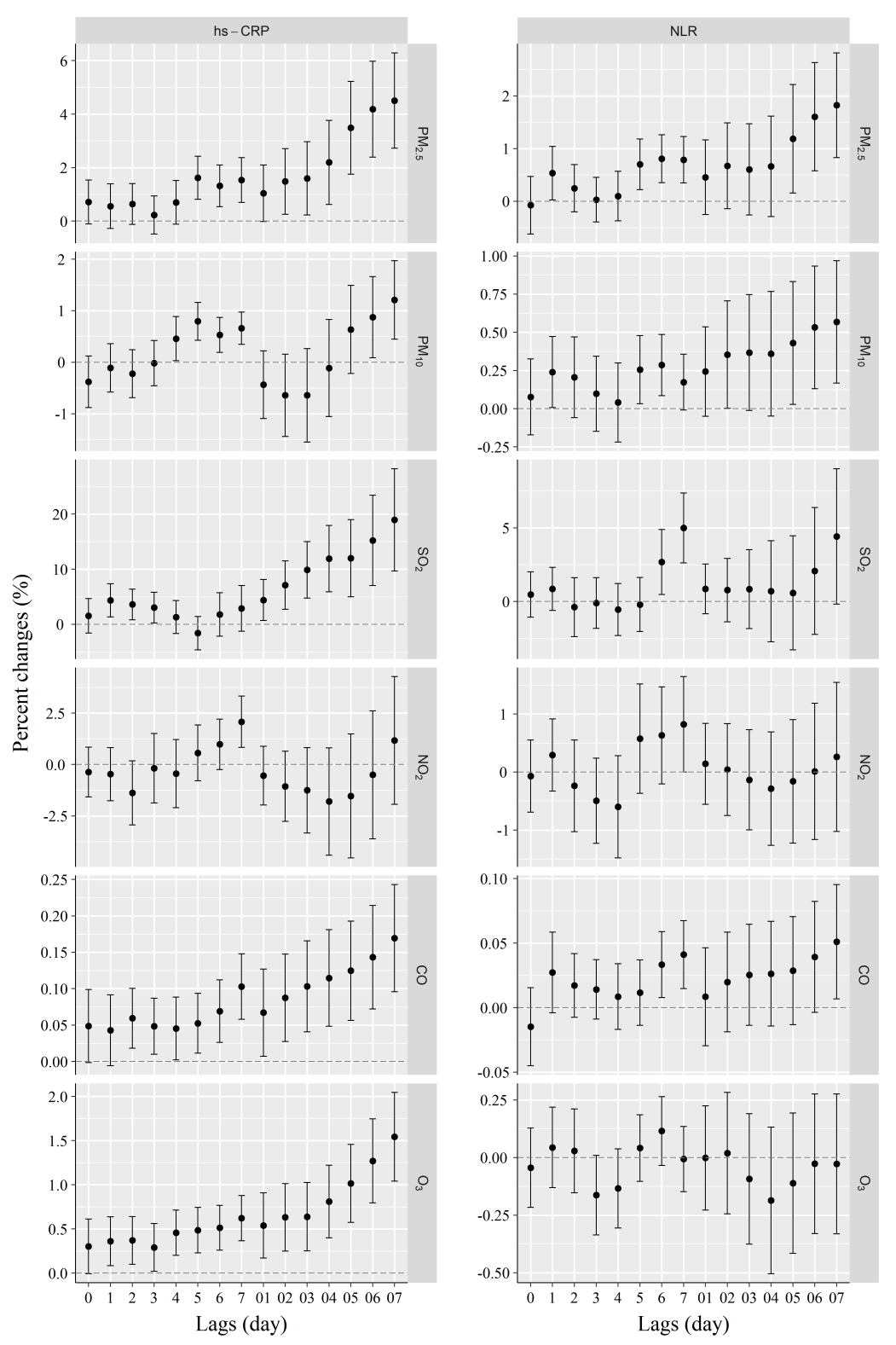


**Fig. 3**

**
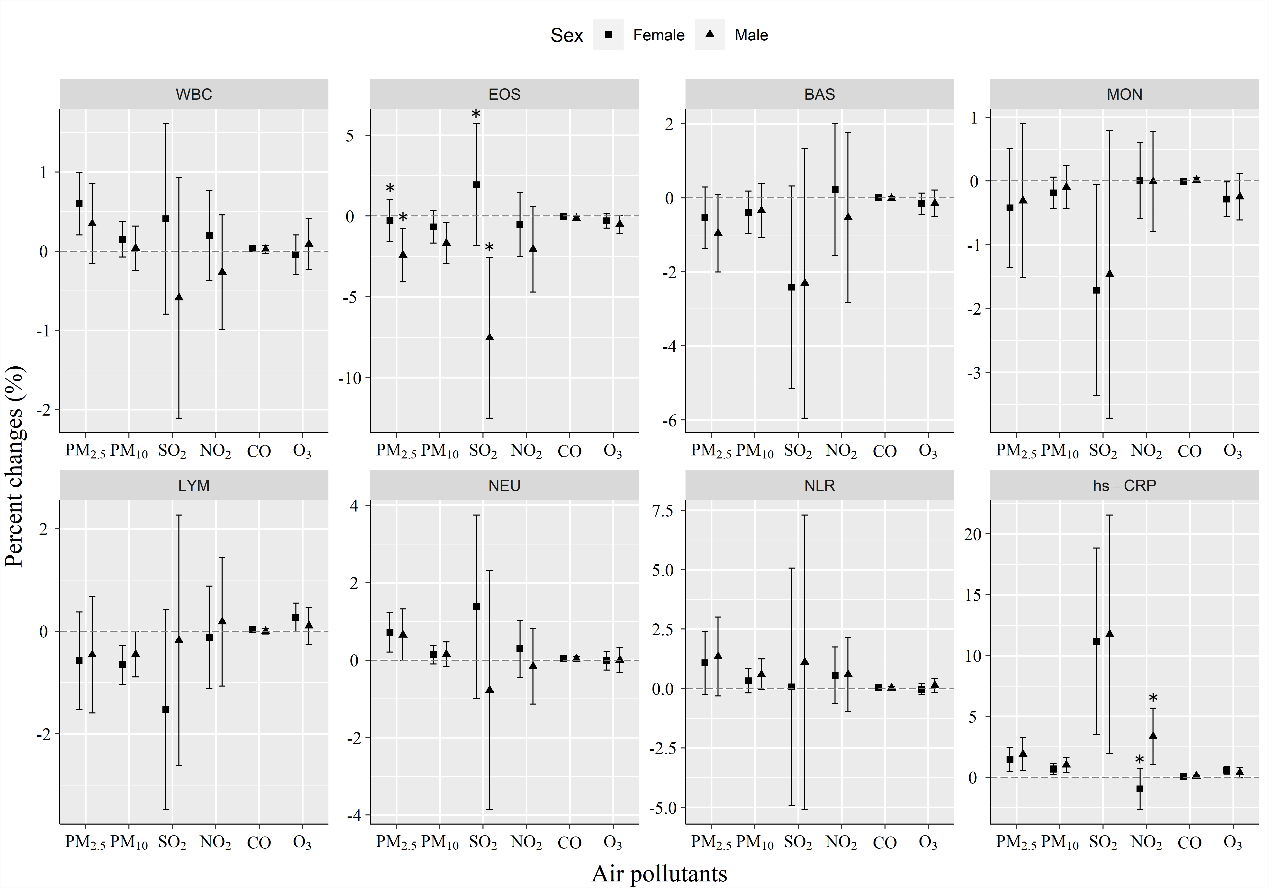
**

**Fig. 4**


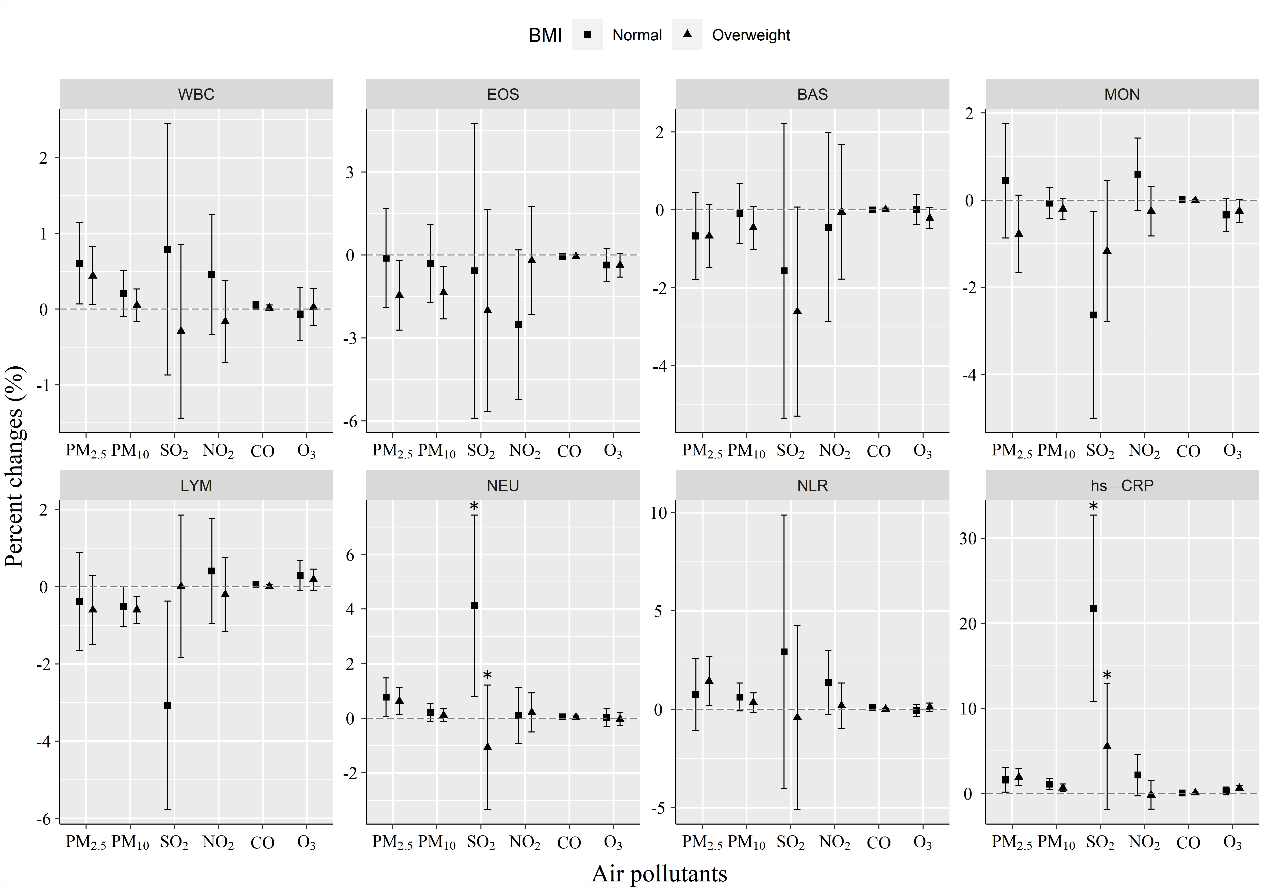


**Fig. 5**


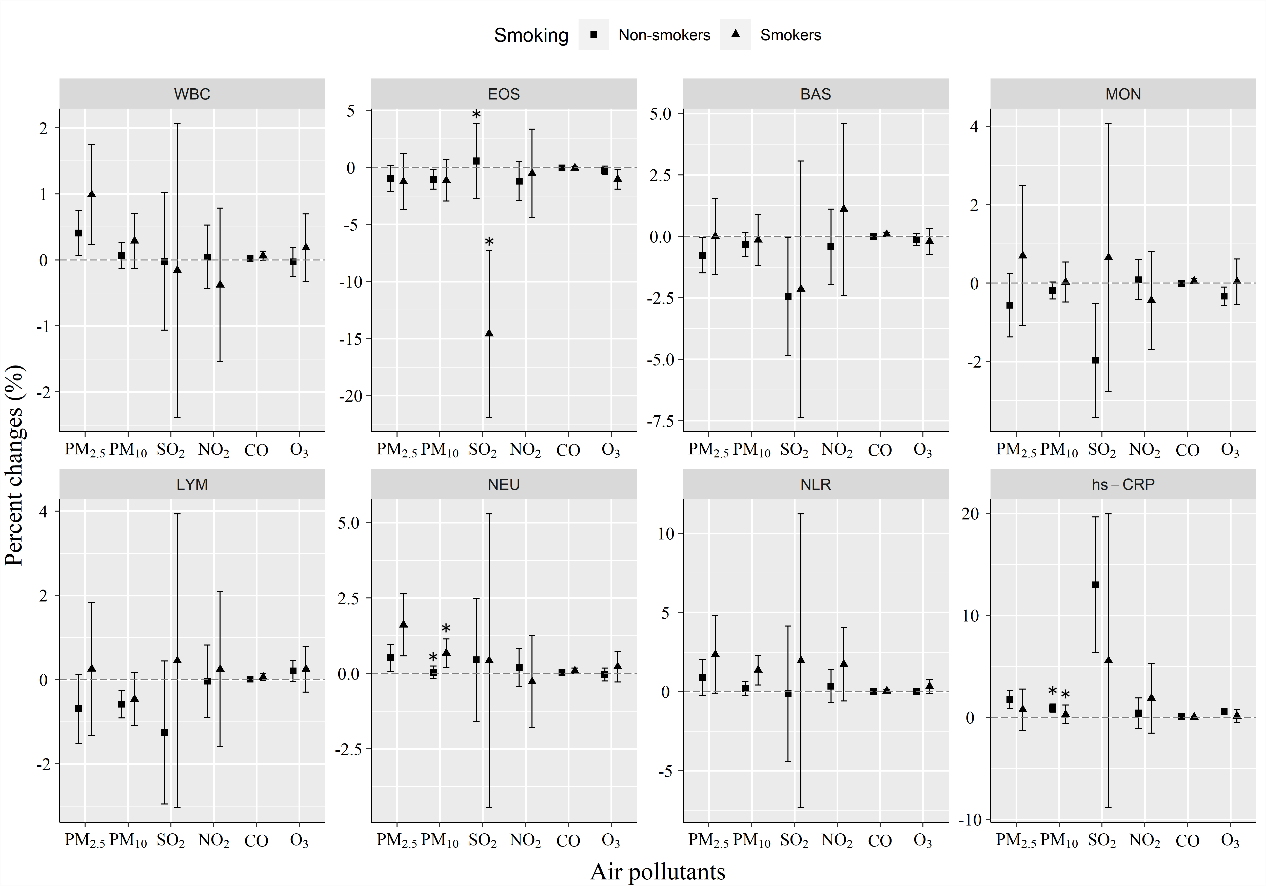


**Fig. 6**

**
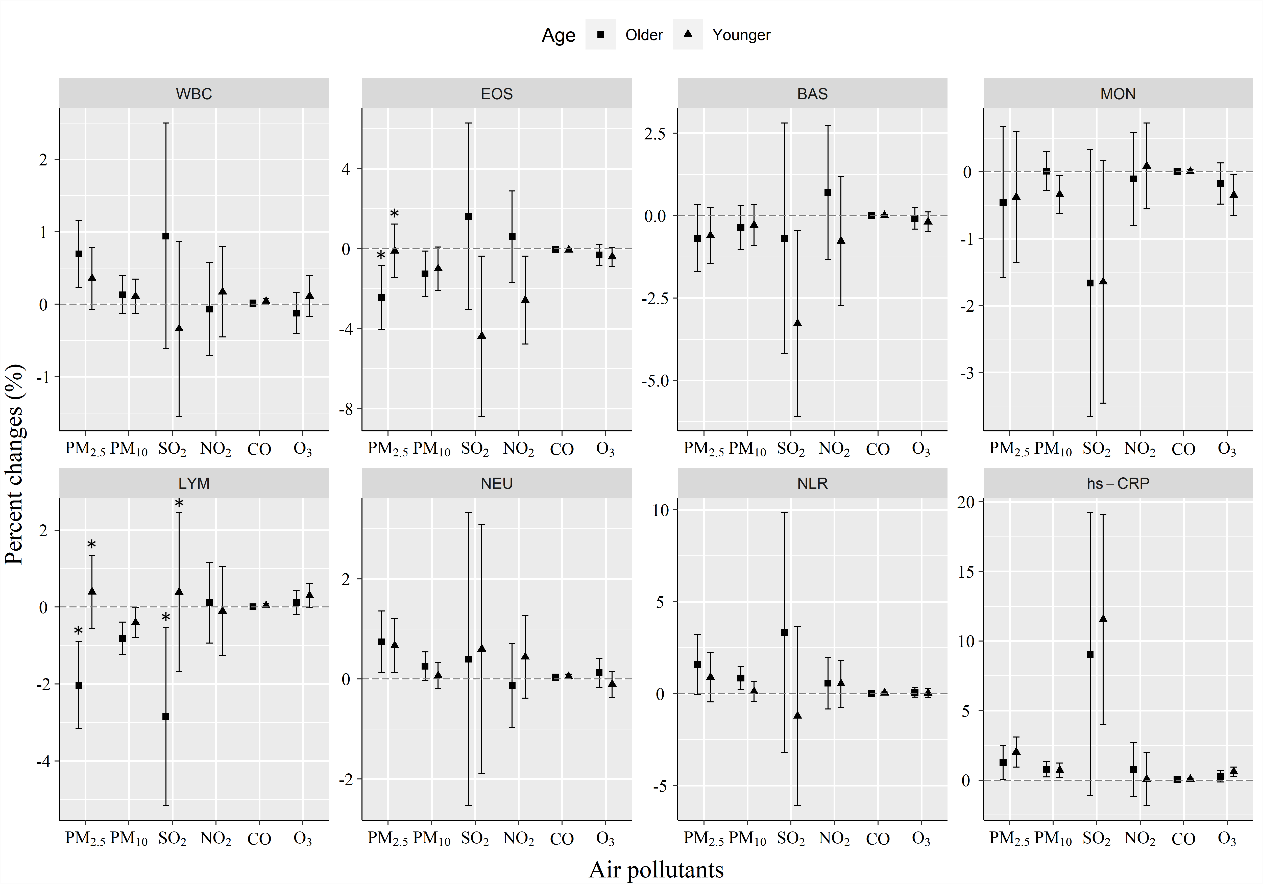
**

| **Table 3: Percent Changes (95% CI) of immunological and inflammatory biomarkers with a 10 μg/m^3^ increase in PM_2.5_ in two-pollutant model.** | | | | | | | | | |
| --- | --- | --- | --- | --- | --- | --- | --- | --- | --- |
|  |  | WBC (%) | EOS (%) | BAS (%) | MON (%) | LYM (%) | NEU (%) | hs-CRP (%) | NLR (%) |
| PM_2.5_ | PM_2.5_ | **0.51 (0.19, 0.82)** | **-1.06 (-2.09, -0.04)** | **-0.65 (-1.3, 0)** | -0.35 (-1.09, 0.38) | -0.53 (-1.26, 0.2) | **0.7 (0.29, 1.1)** | **1.62 (0.82, 2.43)** | **1.19 (0.16, 2.22)** |
|  | PM_2.5_+PM_10_ | **0.54 (0.22, 0.99)** | **-1.05 (-2.08, 1.48)** | -0.64 (-1.3, -0.03) | -0.38 (-1.12, -0.28) | -0.49 (-1.27, 0.53) | **0.75 (0.32, 2.43)** | **1.95 (1.11, 17.96)** | **1.17 (0.12, 4.46)** |
|  | PM_2.5_+SO_2_ | **0.51 (0.19, 0.46)** | -1.02 (-2.08, 0.53) | -0.64 (-1.33, 1.29) | -0.35 (-1.08, 0.49) | -0.65 (-1.41, 0.78) | **0.7 (0.29, 0.72)** | **1.62 (0.79, 1.92)** | **1.17 (0.13, 1.52)** |
|  | PM_2.5_+NO_2_ | **0.51 (0.2, 0.06)** | **-1.11 (-2.13, 0)** | **-0.65 (-1.3, 0.03)** | -0.4 (-1.18, 0.02) | -0.55 (-1.3, 0.06) | **0.7 (0.3, 0.08)** | **1.68 (0.87, 0.1)** | **1.4 (0.3, 0.06)** |
|  | PM_2.5_+CO | **0.48 (0.16, 0.2)** | **-1.08 (-2.1, -0.04)** | **-0.72 (-1.39, 0.08)** | -0.49 (-1.24, -0.06) | **-0.75 (-1.49, 0.44)** | **0.7 (0.28, 0.19)** | **1.53 (0.72, 0.74)** | **1.44 (0.37, 0.22)** |
|  | PM_2.5_+O_3_ | **0.49 (0.18, 0.82)** | **-1.06 (-2.08, -0.05)** | **-0.75 (-1.41, -0.07)** | -0.28 (-1.02, 0.39) | -0.43 (-1.17, -0.16) | **0.69 (0.28, 1.15)** | **1.57 (0.76, 2.36)** | **1.18 (0.15, 2.37)** |
| The statistically significant estimates are highlighted in bold.  WBC: white blood cells; EOS: eosinophils; BAS: basophils; MON: monocytes; LYM: lymphocytes; NEU: neutrophils; hs-CRP: high-sensitivity C-reactive protein. | | | | | | | | | |
